# Identification of harmful cyanobacteria in the Sacramento-San Joaquin Delta and Clear Lake, California by DNA barcoding

**DOI:** 10.1186/2193-1801-2-491

**Published:** 2013-09-30

**Authors:** Tomofumi Kurobe, Dolores V Baxa, Cécile E Mioni, Raphael M Kudela, Thomas R Smythe, Scott Waller, Andrew D Chapman, Swee J Teh

**Affiliations:** Department of Anatomy, Physiology, and Cell Biology, School of Veterinary Medicine, University of California, Davis, CA 95616 USA; Institute of Marine Sciences, University of California, Santa Cruz, CA 95064 USA; Lake County Water Resources Department, Lakeport, CA 95453 USA; California Department of Water Resources, Environmental Monitoring Program, West Sacramento, CA 95691 USA; Greenwater Laboratories, Palatka, FL 32177 USA

**Keywords:** Harmful cyanobacteria, DNA barcoding, 16S ribosomal DNA, Internal transcribed spacer region

## Abstract

Accurate identification of cyanobacteria using traditional morphological taxonomy is challenging due to the magnitude of phenotypic plasticity among natural algal assemblages. In this study, molecular approach was utilized to facilitate the accurate identification of cyanobacteria in the Sacramento-San Joaquin Delta and in Clear Lake in Northern California where recurring blooms have been observed over the past decades. Algal samples were collected from both water bodies in 2011 and the samples containing diverse cyanobacteria as identified by morphological taxonomy were chosen for the molecular analysis. The 16S ribosomal RNA genes (16S rDNA) and the adjacent internal transcribed spacer (ITS) regions were amplified by PCR from the mixed algal samples using cyanobacteria generic primers. The obtained sequences were analyzed by similarity search (BLASTN) and phylogenetic analysis (16S rDNA) to differentiate species sharing significantly similar sequences. A total of 185 plasmid clones were obtained of which 77 were successfully identified to the species level: *Aphanizomenon flos-aquae, Dolichospermum lemmermannii* (taxonomic synonym: *Anabaena lemmermannii*)*, Limnoraphis robusta* (taxonomic synonym: *Lyngbya hieronymusii* f. *robusta*) and *Microcystis aeruginosa*. To date, *Dolichospermum* and *Limnoraphis* found in Clear Lake have only been identified to the genus lavel by microscopy. During the course of this study, morphological identification and DNA barcoding confirmed *A. flos-aquae* as the predominant cyanobacterium in the Sacramento-San Joaquin Delta indicating a shift from *M. aeruginosa* that have dominated the blooms in the past decade. Lastly, the species-specific identification of *Limnoraphis robusta* in Clear Lake is another significant finding as this cyanobacterium has, thus far, only been reported in Lake Atitlan blooms in Guatemala.

## Background

Harmful cyanobacterial blooms (CyanoHABs) are a serious global concern and are often associated with odorous metabolites in drinking water and toxins in aquaculture facilities and in the environment (Mankiewicz et al. 2003; Smith et al. 2008). Different types of toxins are produced from several cyanobacterial species including hepatotoxins (microcystins), cytotoxins (cylindrospermopsin), neurotoxins (anatoxin-a, antillatoxin, saxitoxins), and dermatoxins (lyngbyatoxins). These potent toxins render serious consequences to the health of ecosystems, aquatic organisms, domestic animals, and humans upon direct contact or consumption of CyanoHAB impacted water (Mankiewicz et al. 2003; Osswald et al. 2007; Puschner et al. 2010; Acuña et al. 2012).

The current study focused on molecular analysis of cyanobacterial species from two ecosystems that are ecologically and economically important in California. The Sacramento-San Joaquin Delta is a critical water supply system in Northern California, which provides drinking water to two-thirds of the California population (more than 20 million people) and irrigates 4.5 million acres of farmlands (Jassby 2008). The estuary also provides essential habitats for many anadromous, commercial, and recreational fish such as striped bass (*Morone saxatilis*), Chinook salmon (*Oncorhynchus tshawytscha*), and several endangered fish species such as the delta smelt (*Hypomesus transpacificus*) (Sommer et al. 2007). Blooms of the hepatotoxin-producing cyanobacterium *Microcystis aeruginosa* were first recorded in the Sacramento-San Joaquin Delta in 1999, and since then, cyanobacterial blooms have re-occurred and have been monitored for biomass and toxicity (Lehman et al. 2005, 2008, 2010; Spier et al. 2010). Colonial forms of *M. aeruginosa* are widely distributed along the 180 km of freshwater and brackish waterways of the delta that may affect indigenous invertebrates and fishes (Lehman et al. 2005, 2008, 2010; Ger et al. 2010). Other harmful cyanobacteria such as *Aphanizomenon, Dolichospermum* (formerly recognized as planktic *Anabaena*) (Wacklin et al. 2009), and *Oscillatoria* have been observed in the Sacramento-San Joaquin Delta, although to a lesser extent than *Microcystis* (Cloern and Dufford 2005; Lehman et al. 2010; Spier et al. 2010). Because algal bloom studies in the Delta have mainly focused on *M. aeruginosa* (Lehman et al. 2005, 2008, 2010), the occurrence, abundance, and potential role of other toxin-producing cyanobacteria to indigenous fisheries resources are largely unknown.

Clear Lake is the largest natural lake in California and provides drinking water to local communities. The lake supports recreational activities and tourism for sport fishing and water contact sports, forming an industry greater than 50 million dollars in the local county (Goldstein and Tolsdorf 1994). Although considered “impaired” in terms of hyper-eutrophication from phosphorus and sulfate overload from anthropogenic activities, the lake is used for storage of irrigation water for downstream agricultural lands. Land use such as construction of farmlands, road building, livestock grazing, logging, and firewood cutting have accelerated erosion resulting in large phosphorus inputs mostly from basins around the lake (Richerson et al. 1994). Cyanobacterial assemblages in the lake reached the highest densities from the mid 1970’s to 1990 (Horne 1975; Richerson et al. 1994; Winder et al. 2010) and were dominated by diazotrophic cyanobacteria such as *Aphanizomenon*, *Dolichospermum*, and the non-nitrogen fixing cyanobacterium *Microcystis* (Horne 1975; Richerson et al. 1994). *Lyngbya* (now known as *Limnoraphis*) blooms have also been recorded since 2009 (Mioni and Kudela 2011, Mioni et al. 2012). These cyanobacteria form scum on the water surface and deteriorate water quality (Smith et al. 2008; Mioni and Kudela 2011, Mioni et al. 2012).

Cyanobacteria are traditionally classified and identified by microscopic analysis of morphological characters such as shape and size of vegetative cells, heterocytes, akinetes, presence/absence of sheath, and morphology of terminal cell. This task is challenging even for a well-experienced taxonomist due to significant phenotypic changes that may occur in natural assemblages and morphological transformation upon cultivation in the laboratory environment (Palinska et al. 1996). Comprehensive morphological identification combined with molecular characteristics have been reported for cyanobacteria found in Nordic countries belonging to the order Nostocales such as *Anabaena*, *Aphanizomenon*, *Dolichospermum*, *Trichormus*, and *Nostoc* (Rajaniemi et al. 2005; Wacklin et al. 2009). Genetic relationships have been characterized among Chroococcales (*Cyanobium, Synechocystis*, and *Synechococcus*), Oscillatoriales (*Leptolyngbya, Microcoleus, Phormidium*, and *Romeria*), and Nostocales (*Nostoc* and *Nodularia*) in Portuguese estuaries (Lopes et al. 2012). These studies have greatly enriched the cyanobacterial database by linking genetic information and morphological features to facilitate species identification.

DNA barcoding is a taxonomic identification method that relies on the use of standardized species-specific DNA regions known as “barcodes” (Hebert et al. 2003). Species identification by DNA barcodes provides a rapid and specific detection tool for various organisms such as mammals (Murphy et al. 2001), birds (Khan et al. 2010), amphibians (SanMauro et al. 2005), and fish (Kochzius et al. 2010). Because each organism possesses unique gene sequences, DNA barcoding offers an accurate identification of known species and leads to the discovery of unique organisms with discrete genetic profiles. DNA barcoding has been employed for assessment of cyanobacterial assemblages (Betournay et al. 2007; Lopez-Legentil et al. 2011) and genetic diversity of diatoms and dinoflagellates (Litaker et al. 2007; Lin et al. 2009; Moniz and Kaczmarska 2010). DNA barcoding has also been used to analyze changes in bacterial community composition potentially affecting biotic interactions due to *Microcystis* blooms (Cheng et al. 2011).

Over the last decades, the species composition of recurring blooms in the Sacramento-San Joaquin Delta and Clear Lake has been assessed by traditional morphological taxonomy. As morphological identification is not always conclusive, molecular analysis such as sequencing of species-specific regions followed by phylogenetic analysis is a widely applied technique for obtaining precise taxonomic classification of biological specimens (Robertson et al. 2001; Casamatta et al. 2005; Rajaniemi et al. 2005; Ezhilarasi and Anand 2009; Lopes et al. 2012). Our goal in the current study is to facilitate the accurate identification of dominant cyanobacterial species from two water bodies in California impacted by seasonal CyanoHABs by traditional taxonomic identification combined with molecular techniques.

## Results

### Microscopy

Microscopic observation of samples collected in Clear Lake showed four filamentous (*Aphanizomenon* spp., *Dolichospermum* (formerly *Anabaena*) spp., *Limnoraphis* (formerly *Lyngbya*) spp., and *Gloeotrichia echinulata*) and two colonial (*M. aeruginosa* and *Woronichinia naegeliana*) cyanobacteria (Table 1). Although the samples from the Sacramento-San Joaquin Delta showed that *Aphanizomenon* spp., *Dolichospermum* spp., and *M*. *aeruginosa* were dominant as observed by microscopy, other cyanobacterial species such as *Limnoraphis*, *Gloeotrichia*, and *Woronichinia* that were found in Clear Lake were not observed in the Delta by traditional microscopy (Table 1). As briefly mentioned above, all planktic morphospecies in the genus *Anabaena* have been transferred into the new genus *Dolichospermum* (Wacklin et al. 2009). Likewise, a tropical planktic filamentous cyanobacteria found only in Lake Atitlan, Guatemala, formerly identified as *Lyngbya*, has been classified into a new genus, *Limnoraphis* (Komárek et al. 2013).

**Table 1:** **Algal samples used for molecular analysis**

Location	Sample	APHN	DLCH	GLTR	LMNR	MCRC-AE	WRNC
	ID	(filmt/mL)	(cells/mL)	(filmt/mL)	(filmt/mL)	(cell/mL)	(cell/mL)
Clear Lake	CL3(6)^a^	54,950	312,430	136,982	2,355	819,540	ND
		(4.1%)	(23.6%)	(10.3%)	(0.2%)	(61.8%)	
	CL4(7)	ND	ND	648,606	15,700	2,219,587	ND
				(22.5%)	(0.5%)	(77.0%)	
	S2(7)	ND	516,137	1,018,537	406,237	ND	ND
			(26.6%)	(52.5%)	(20.9%)		
	S1(8)	245	179,814	25,267	10,058	ND	ND
		(0.1%)	(83.5%)	(11.7%)	(4.7%)		
	CL1(9)	ND	132,273	ND	5,888	153,467	547,538
			(15.8%)		(0.7%)	(18.3%)	65.2%
Sacramento-	D16(7)	ND	41,998	ND	ND	ND	ND
San Joaquin			(100%)				
Delta	D16(9)	41,409	ND	ND	ND	38,858	ND
		(51.6%)				(48.4%)	
	D19(9)	70,846	2,551	ND	ND	ND	ND
		(96.5%)	(3.5%)				

The species composition of cyanobacterial species, indicated as percentage, was determined by morphological identification (Mioni et al. 2012).

*Abbreviations*: *APHN* *Aphanizomenon* spp.: *DLCH* *Dolichospermum* spp.: *GLTR* *Gloeotrichia* spp.: *LMNR* *Limnoraphis* spp.: *MCRC-AE* *Microcystis aeruginosa*: *WRNC* *Woronichinia* spp: *ND* not detected.

^a^The sampling month is shown in parenthesis in Sample ID.

### Molecular analyses

We obtained a total of 185 clones showing similarity to sequences of potentially toxin-producing cyanobacteria including *Aphanizomenon, Dolichospermum, Limnoraphis, Microcystis* as well as various types of bacteria such as *Synechococcus, Bacillus, Paenibacillus, Fluviicola,* alpha-proteobacteria*,* and *Rhodobacter* (Table 2). Among the clones, 77 sequences showing similarity to *Aphanizomenon, Dolichospermum, Limnoraphis,* and *Microcystis* were classified into 14 genotypes (Table 3) based on the degree of the similarities of their 16S ribosomal RNA gene (rDNA) and internal transcribed spacer (ITS) sequences using a 98.5% cutoff value as stringent criteria for species identification (Janda and Abbott 2007). The sequences of the type clone for each group were deposited in NCBI GenBank (accession numbers JX006082 to JX006095).

**Table 2:** **Cyanobacteria and other bacteria in the Sacramento-San Joaquin Delta and Clear Lake identified by molecular analyses**

Location	SampleID	APHN-FL	DLCH-LE	DLCH-sp	LMNC-LI	LMNR-RO	LMNR-sp	MCRC-AE	ALGR-sp	aPRTB	BCLL-PU	FLVC-TA	PNBC-AL	PNBC-sp	RHDB-SP	RHDB-sp	SYNC-sp	UNKWN^a^
Clear Lake	CL3(6)^b^	21	2	1	2			6			9						6	
	CL4(7)												14	6				
	S2					1	1		1	12					1	1	2	
	S1(8)					4	2					1					12	
	CL1(9)																20	
Sacramento-San Joaquin Delta	D16(7)^c^																23	2
	D19(9)	20																
	D19(9)	13		7														

Numbers indicate the number of clones classified into each category.

Twenty clones were analyzed for each sample, except for CL3(6) that used 50 clones for sequencing. The sequencing reaction did not work for some of the clones, affecting the total number of clones available in this Table. The obtained 16S rDNA sequences were subjected to clustering into Operational Taxonomic Units and similarity search by BLASTN program. Phylogenetic analysis was conducted to identify closely related taxa.

*Abbreviations:* [cyanobacteria] APHN-FL: *Aphanizomenon flos-aquae*, DLCH-LE: *Dolichospermum lemmermannii*, DLCH-sp: *Dolichospermum* sp., LMNC-LI: *Limnococcus limneticus*, LMNR-RO: *Limnoraphis robusta*, LMNR-sp: *Limnoraphis*-sp., MCRC-AE: *Microcystis aeruginosa*, [other bacteria] ALGR-sp: *Algoriphagus* sp., aPRTB: alpha proteobacterium, BCLL-PU: *Bacillus pumilus*, FLVC-TA: *Fluviicola taffensis*, PNBC-AL: *Paenibacillus alvei*, PNBC-sp: *Paenibacillus* sp., RHDB-SP: *Rhodobacter sphaeroides*, RHDB-sp: *Rhodobacter* sp., SYNC-sp: *Synechococcus* sp., UNKWN: unknown bacteria

^a^These two sequences were designated as unidentified bacteria (maximum identity by BLASTN search <95%).

^b^The sampling month is shown in parenthesis in Sample ID.

^c^Different primer set (CYA108F and CYA16S SCYR) was used for the sample D16(7).

**Table 3:** **Defined genotypes of cyanobacteria identified from the Sacramento-San Joaquin Delta and Clear Lake**

Genotype ID	Classification	Accession number
SWMP11-01	*Aphanizomenon flos-aquae*	JX006082
SWMP11-02	*A. flos-aquae*	JX006083
SWMP11-04	*A. flos-aquae*	JX006085
SWMP11-07	*A. flos-aquae*	JX006088
SWMP11-08	*A. flos-aquae*	JX006089
SWMP11-06	Unknown, belong to Nostocaceae	JX006087
SWMP11-03	*Dolichospermum* sp.	JX006084
SWMP11-05	*D. lemmermannii*	JX006086
SWMP11-09	*Microcystis aeruginosa*	JX006090
SWMP11-10	*M. aeruginosa*	JX006091
SWMP11-11	*M. aeruginosa*	JX006092
SWMP11-12	*Limnoraphis robusta*	JX006093
SWMP11-13	*Limnoraphis* sp.	JX006094
SWMP11-14	*Limnoraphis* sp.	JX006095

BLAST search and phylogenetic analysis were utilized for the classification.

**Figure 1:**
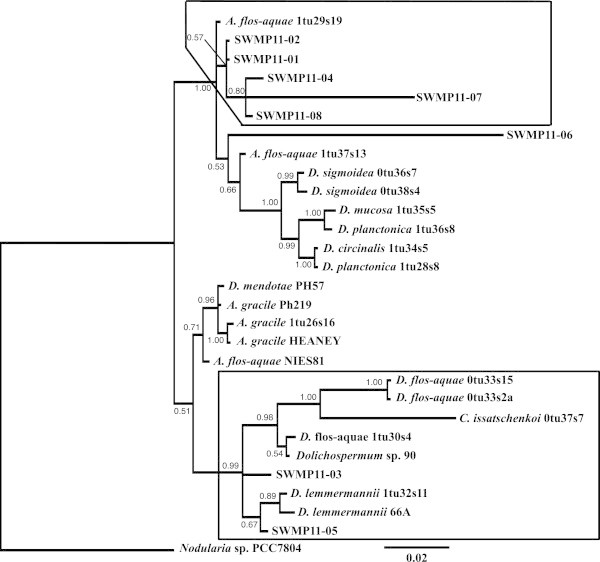
**Phylogenetic tree for the genotypes identified from the Sacramento-San Joaquin Delta and Clear Lake (SWMP11-01 to -08).** The 16S rDNA sequences for *Aphanizomenon*, *Cuspidothrix*, and *Dolichospermum* species described in Rajaniemi et al. (2005) and Wacklin et al. (2009) were used for the analysis. The boxes indicate the clades including the genotypes obtained in this study. Number at each node represents the posterior probability value. The scale bar indicates inferred number of substitutions. *Nodularia* sp. (strain PCC7804) sequence was used as the outgroup.

Although BLASTN search is a commonly used and powerful tool for similarity analysis, it is incapable of distinguishing species that share very similar gene sequences. For example, *Aphanizomenon*, *Anabaena*, and *Dolichospermum* share significantly high similarity scores (>98%) in the 16S rDNA sequence rendering an inconclusive molecular identification. This difficulty was addressed by constructing phylogenetic trees as depicted in this study, providing an accurate identification of the cyanobacterial species. We obtained over 40 clones showing sequence similarity to either *Aphanizomenon* or *Dolichospermum* by BLASTN search from both the Sacramento-San Joaquin Delta and Clear Lake. Phylogenetic analysis successfully classified the majority of the sequences as *Aphanizomenon (A.) flos-aquae* (Figure 1). The genotypes, SWMP11-01, -02, -04, -07, and -08 formed a clade with morphologically identified *A. flos-aquae* strain 1tu29s19 as described in Rajaniemi et al. (2005); Wacklin et al. (2009), suggesting that these clones are highly likely amplified from *A. flos-aquae*. Likewise, the classification of the genotypes SWMP11-05 showing similarity to *Dolichospermum* (*D.*) *lemmermannii* that were amplified from algal samples collected in Clear Lake were also confirmed (Figure 1).

**Figure 2:**
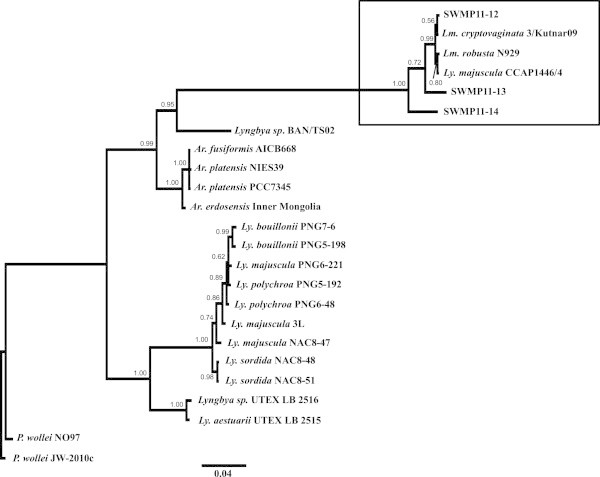
**Phylogenetic tree for the genotypes obtained from Clear Lake.** Algal samples (SWMP11-12 to -14) were compared with closely related taxa, *Arthrospira* (*Ar.*), *Lyngbya* (*Ly.*), and *Limnoraphis* (*Lm.*). The 16S rDNA sequences were used for depicting the tree. The box indicates the clade including the genotypes from this study. Number at each node represents the posterior probability value. The scale bar indicates inferred number of substitutions. Sequences from *Plectonema (P.) wollei* strain JW-2010c was used as the outgroup.

Three of the genotypes, SWMP11-12 to -14 were placed in a clade with *Limnoraphis* (*Lm.*) *cryptovaginata*, *Lm. robusta,* and *Lyngbya* (*Ly.*) *majuscula* in Figure 2. Notably, 16S rDNA sequence of SWMP11-12 is almost identical to those of *Lm. cryptovaginata*, *Lm. robusta*, and *Ly. majuscula* with a few base differences in a 1.5 kb sequence (>99.8%). The other genotypes, SWMP11-13 and -14 were also placed in the same clade, however, these sequences are distinct from any sequences in GenBank Database (16S rDNA, <95%).

## Discussion

Using a combination of similarity search (BLASTN) and phylogenetic analysis, several species of cyanobacteria were identified in Clear Lake including *Aphanizomenon flos-aquae*, *Dolichospermum* (formerly *Anabaena*) *lemmermannii*, *Dolichospermum* spp., *Limnoraphis* (formerly *Lyngbya*) *robusta*, *Microcystis aeruginosa*, and *Synechococcus* spp. In contrast, fewer species of cyanobacteria were observed in the Sacramento-San Joaquin Delta that included *A. flos-aquae*, *Dolichospermum* spp., and *Synechococcus* spp. Other species of *Microcystis* were not observed in the samples examined from the Sacramento-San Joaquin Delta and Clear Lake by molecular analysis. Of particular importance is the species-specific identification of *A. flos-aquae*, *D. lemmermannii*, and *Lm. robusta* in Clear Lake; to date, they (especially *D. lemmermannii*, and *Lm. robusta*) have been identified only to the genus level by morphological analysis. In addition, DNA barcoding identified a small nondescript unicellular cyanobacterium as *Synechococcus* spp. from both Clear Lake and the Sacramento-San Joaquin Delta. Further analysis such as isolation, microscopy, and sequencing are necessary to determine their species-specific identification. In addition to cyanobacteria, other bacteria were also present in the samples examined by molecular techniques. For example, Gram-positive (i.e. *Paenibacillus alvei* and *Bacillus pumilus*) and Gram-negative (i.e. alpha proteobacteria and Flexibacteria) bacteria were also found in the bloom samples examined from Clear Lake. While these bacteria can tolerate various ranges of environmental conditions and are expected to be present in the water column, their potential role in algal blooms have not been extensively studied.

*Aphanizomenon flos-aquae* was the dominant species from the 2011 blooms in the Sacramento-San Joaquin Delta as determined by DNA barcoding and by morphological identification (Tables 1, 2). In addition, quantitative (q) PCR assays that we designed for *A. flos-aquae* and *M. aeruginosa* based on the obtained sequences in this study revealed more than 2 order of magnitude greater for the *A. flos-aquae* during the 2011 bloom season (unpublished data). While previously observed in less frequency and distribution, blooms of *A. flos-aquae* have not been reported in the Sacramento-San Joaquin Delta in the past decade to the best of our knowledge. Pioneering studies on algal blooms in the Delta showed *M. aeruginosa* as the dominant species in recurring blooms (Lehman et al. 2005, 2008, 2010). Although the cause(s) in the shifts of cyanobacterial assemblages, at least during the duration of this study, in the upper San Francisco Estuary remains unclear, continuous monitoring of dominant and potentially toxin-producing cyanobacteria including *M. aeruginosa* and *A. flos-aquae* is warranted in terms of mitigating their adverse impacts to aquatic organisms and conservation of water quality. Water temperature and other physicochemical factors have been associated with the emergence of *A. flos-aquae* (Cloern and Dufford 2005) and other cyanobacterial species during the 2011 blooms in the Sacramento-San Joaquin Delta (Mioni et al. 2012). *A. flos-aquae* is a diazotrophic cyanobacterium which produce endotoxins such as anatoxin-a, saxitoxins, and cylindrospermopsin (Sivonen and Jones 1999; Castle and Rogers 2009).

A species identification system using a combination of BLASTN search and phylogenetic analysis for 16S rDNA is a powerful method; however this approach has limitations when used to analyze taxa with nearly identical sequences or to classify unknown sequences. For example, *Lm. robusta*, *Lm. cryptovaginata*, and *Ly. majuscula* share identical 16S rDNA sequences (1.5 kb) with a few base pairs differences as observed from 6 clones (SWMP11-12) amplified from Clear Lake samples (Jüttner and Watson 2007; Guiry and Guiry 2012). Although the phylogenetic tree (Figure 2) implies that these clones are most closely related to *Lm. cryptovaginata*, SWMP11-12 is most likely *Lm. robusta* by virtue of their characteristic morphological features (Komárek 2003, Komárek et al. 2013; Rejmánková et al. 2011). While *Ly. majuscula* is also placed in the same clade, SWMP11-12 is unlikely *Ly. majuscula* as it is a marine species (Jones et al. 2011)*. Lm. robusta* blooms in Lake Atitlan, Guatemala was first reported in 2008 by (Rejmánková et al. 2011). This cyanobacterium formed dense patches covering approximately 40% of Lake Atitlan surface during the peak of the blooms although cyanotoxin production from this species remains unclear (Rejmánková et al. 2011). Cylindrospermopsin and saxitoxins were detected during the Atitlan bloom in 2009, but the concentrations remained low (12 and 58 ng g^-1^ from the freeze dried specimen) (Rejmánková et al. 2011; Komárek et al. 2013). *Lm. robusta* found in Clear Lake, California is unlikely introduced from Lake Atitlan as there are no reports documenting the introduction of this species across the two water bodies. Further analysis such as comparison of variable gene region will provide a better understanding of the relationship between the same species found in two distant locations. Initial findings showed that environmental factors such as water temperature and nutrient concentrations may affect the growth and abundance of *Lm. robusta* and other emerging cyanobacteria in Clear Lake during the 2011 blooms (Mioni et al. 2012).

The classification of clone SWMP11-06 in the phylogenetic tree is debatable (Figure 1). The genotype is placed in a clade of *Dolichospermum*, however, the reference strain *A. flos-aquae* strain 1tu37s13, which was morphologically identified by Rajaniemi et al. (2005), is also placed in the same group. The values of posterior probability are relatively low for the branching (< 0.70), precluding a conclusive identification of the genotype.

SWMP11-13 and -14 from Clear Lake showed similarity to *Lm. robusta* by BLASTN search but interestingly these sequences are distinct from *Lm. robusta*, *Lm. cryptovaginata*, or any other sequences in the NCBI-GenBank database (16S rDNA Pairwise% Identity: 96.8%). Stackebrandt and Goebel (1994) suggested a cutoff value of 97.5% (or higher) for acceptable similarity values for species identification using the 16S rDNA sequence. These two genotypes are tentatively designated as *Limnoraphis* sp. as placed in the phylogenetic tree; further analysis is necessary for species-specific identification (Figure 2).

Although *M. aeruginosa* was detected at different sampling sites and times in the Sacramento-San Joaquin Delta by microscopic observation, samples that were examined by molecular analysis did not detect this species. This result may be due to the following reasons: 1) *M. aeruginosa* was lacking in the samples examined for molecular analysis due to its colonial nature and heterogeneity across subsamples, and 2) inhibition by other abundant cyanobacteria precluding the amplification of *M. aeruginosa* in the samples that were PCR tested. It is important to note that *A. flos-aquae*, instead of the historically recurring *M. aeruginosa*, dominated the blooms in the Sacramento-San Joaquin Delta during the duration of these studies. Although the field samples that we chose for molecular analyses were based on morphologic microscopic analysis (qualitative and quantitative), the DNA fragments of the expected algal species were not obtained from the subsamples. Another potential explanation may be due to the small number of clones that were analyzed that may not represent the wide variety of cyanobacterial species present in the blooms. Analyzing more clones from appropriate field samples using emerging sequencing technologies would probably yield a greater number of sequences from potentially toxin-producing cyanobacteria with a sample such as CL3(6) from Clear Lake that showed more diverse bacterial species (Table 2). Another ideal approach is to use parallel algal samples for morphologic taxonomic identification and molecular analyses to better understand the cyanobacterial composition in the Sacramento-San Joaquin Delta and Clear Lake.

Most of the cyanobacterial sequences deposited in NCBI-GenBank database originate from geographically distant locations such as Portugal, Japan, India, and Nordic countries (Robertson et al. 2001; Rajaniemi et al. 2005; Ezhilarasi and Anand 2009; Lopes et al. 2012). Despite the distant origins of species-specific sequences, we were able to successfully identify the taxonomic classification of the clones based on the 16S rDNA. Using the 16S rDNA for classification and identification of cyanobacteria is widely accepted because 1) the gene is present in all bacterial genomes, and 2) the frequency of sequence variations and insertions in this gene serves as a molecular clock and reflects evolutional history, allowing the distinction of a broad range of taxonomic groups and identification of individual species (Casamatta et al. 2005; Janda and Abbott 2007). Although the resolution of the 16S rDNA for specific identification remains debatable due to the high degree of their sequence conservation (Janda and Abbott 2007), the gene has been a reliable barcode providing identification to the genus level, in some cases to specific species level as we have demonstrated in this study. We attempted to use the ITS region in our analysis, however, the sequences were not suitable for alignments due to their high variability when compared with the reference strains from other geographic locations (unpublished observation). Another gene involved in nitrogen fixation, *nif*H, has been used as an alternate barcode for classification and identification of cyanobacteria by providing better resolution for species identification (Zehr et al. 1997). However, the *nif*H gene is not appropriate for analyzing complex algal assemblages by DNA barcoding as non-diazotrophic cyanobacteria such as *M. aeruginosa* do not possess this gene in their genome.

## Conclusion

Microscopic observation coupled with DNA barcoding effectively identified cyanobacterial species in the Sacramento-San Joaquin Delta and in Clear Lake. For the first time in Northern California, this tiered approach provided species-specific identification of dominant species in the blooms including *Microcystis aeruginosa*, *Aphanizomenon flos-aquae*, *Dolichospermum* (formerly *Anabaena*) *lemmermannii*, *Dolichospermum* spp., *Limnoraphis* (formerly *Lyngbya*) *robusta*, *Limnoraphis* spp., and *Synechococcus* spp. The precise identification using DNA barcoding provides two important ecological implications in these water bodies. First, we have identified *A. flos-aquae* as the new dominant species in the Sacramento-San Joaquin Delta during the course of this study, an apparent shift from *M. aeruginosa* that have dominated the recurring blooms at the delta in the past decade. Second, DNA barcoding documented the first occurrence of *Lm. robusta* in North America. To date, this harmful cyanobacterium has only been reported from Lake Atitlan in Guatemala where the climate is different from that in California (Komárek et al. 2013). It is important to understand the factors affecting the emergence of *Lm. robusta* in California and the potential link promoting the growth of the cyanobacterium between the two geographically distant water bodies. Lastly, the identification of prokaryote assemblages by DNA barcoding will enhance the current cyanobacterial monitoring efforts by allowing us to develop specific quantitative PCR (qPCR) assays using the sequences obtained in this study. We are currently validating the reliability and reproducibility of the qPCR tests for estimating the abundance of key cyanobacterial species with potential toxin production. Assessment of cyanobacterial assemblages using the interdisciplinary approach (i.e. DNA barcoding and qPCR supported by morphological identification) will aid in formulating effective mitigation measures by addressing the specific identity of cyanobacteria, their corresponding physiological features, and determining the effects of fundamental environmental factors on species-specific toxicity.

## Materials and methods

### Study sites and collection of algal samples

**Figure 3:**
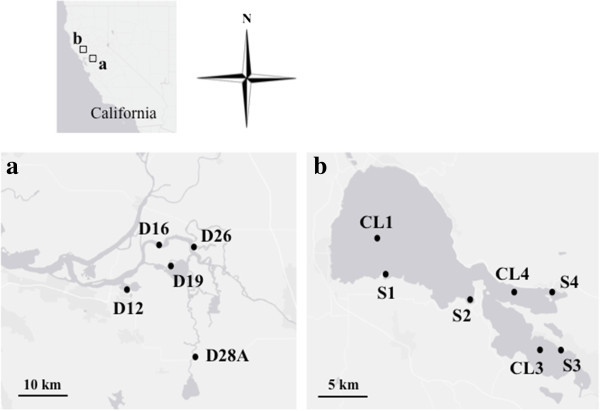
**Sampling locations in the Sacramento-San Joaquin Delta (a) and Clear Lake (b).** Algal samples were collected from 5 stations in the Sacramento-San Joaquin Delta and 7 stations in Clear Lake from June to October in 2011 as part of a monitoring program.

Algal samples were collected for the period of June to October in 2011 from five and seven stations in the Sacramento-San Joaquin Delta and Clear Lake, respectively (Figure 3). These sampling stations have been previously established by the Department of Water Resources as standard monitoring sites with corresponding environmental data such as water quality, nutrient loading, and phytoplankton records (Richerson et al. 1994; Winder et al. 2010). Algal samples were collected according to standard protocol (Fetscher et al. 2009) and established procedures (Mioni and Kudela 2011). Briefly, samples for microscopy were fixed with 2.5% (v/v) glutaraldehyde in the field and were filtered through a 1-μm pore size, 25-mm diameter, black polycarbonate filters (GE Osmonics, Monroe, NC). Algal samples for molecular analysis were collected as follows: approximately 600 mL of surface water (grab) samples were filtered with a 0.45-μm membrane using a clean filtration device (hand pump) on site. Each filter was placed in a sterile microcentrifuge tube and stored on dry ice and in the dark upon collection and transported to the lab for analysis. The samples were stored in a freezer (-80°C) until processing.

### Microscopic analysis

Algal samples were sent to two independent laboratories: University of California, Santa Cruz, and Greenwater Laboratories (http://greenwaterlab.com/) for morphologic taxonomic identification of cyanobacteria (Karlson et al. 2010; Mioni and Kudela 2011). In UC Santa Cruz, epifluorescence microscopy was used to identify and enumerate cyanobacteria present in environmental samples following established procedures (Mioni and Kudela 2011). The abundance of autofluorescing phycoerythrin containing cells (i.e. cyanobacteria) was determined on a Zeiss Axioplan epifluorescence microscope at 400X magnification using green excitation (Zeiss Filter Set 20, excitation 546 nm bandpass, and emission 575–640 nm bandpass filters). At Greenwater Laboratories, samples were preserved in Lugol’s Iodine solution, and cyanobacterial cells were enumerated on a Nikon Eclipse TE200 inverted microscope as described in Standard Methods (American Public Health Association 1992).

The densities of colonial (*Microcystis* and *Woronichinia*) or filamentous (*Aphanizomenon*, *Dolichospermum*, *Limnoraphis*, and *Gloeotrichia*) cyanobacteria were estimated by counting the number of units within one large grid square using a 400X magnification. Twenty grids per filter were counted for each sample. The presence or absence of nitrogen-fixation cells (heterocysts) was observed for *Aphanizomenon*, *Dolichospermum*, and *Gloeotrichia*. Morphological characteristics were determined by cell shape and changes in autofluorescence or cell organization.

### Molecular analysis for species identification

From a total of 60 water samples obtained from both study sites, cyanobacterial samples from the particular sites and months from the Sacramento-San Joaquin Delta (N = 3) and Clear Lake (N = 5) were chosen for molecular analysis. Samples were selected based on the diversity of species assemblages as determined by morphological identification and cell counts targeting *M. aeruginosa, Aphanizomenon, Dolichospermum,* and *Limnoraphis* as mainly observed in both study sites (Table 1). Algal samples were processed for genomic DNA extraction following a phenol extraction method previously used for cyanobacteria from the San Francisco Estuary (Baxa et al. 2010). Since our major interest is colonial or filamentous cyanobacteria, algal cells were aseptically scraped from each filter membrane using forceps, and then transferred into a 100 μl of lysis buffer (10 mM Tris HCl pH 8.0, 1 mM EDTA, 100 mM NaCl, 0.2% sodium dodecyl sulfate). The filter was rinsed with the lysis buffer to recover most of the remaining algal cells on the filter. After suspension of the algal cells by vortex mixer, proteinase K (50 μg/ml final concentration) was added. The samples were placed in a 50°C shaking incubator until algal cells were completely digested (48–72 hrs). At the end of the extraction procedure, Tris (pH 8.0)-EDTA buffer was added to suspend the genomic DNA, and concentration was measured by Nanodrop spectrophotometer (Thermo Fisher Scientific Inc.).

The 16S rDNA and its adjacent ITS region, a region in a bacterial genome commonly used for species identification (Neilan et al. 1997; Casamatta et al. 2005), was amplified by PCR using the generic primer set pA (Edwards et al. 1989) and B23S (Lepere et al. 2000) as described in Rajaniemi et al. (2005) (Table 4). In addition, another set of primers (CYA 108 F and CYA16S SCYR, Table 4) amplifying a partial fragment of the 16S rDNA, but not the ITS, was used for one of the algal samples, D16(7), from the Sacramento-San Joaquin Delta as we observed inhibition of PCR amplification with the primer set described above. The volume of the PCR cocktail was 50 μl containing 200 μM each of dNTP, 1.5 mM of MgCl_2_, 40 pmol of each primer, 2 units of Taq DNA polymerase (High Fidelity Platinum Taq polymerase, Invitrogen Corp) and 10X buffer at 1/10 the volume of the reaction. Bovine serum albumin (0.1 mg/ml final concentration) was added to the reaction cocktail for the algal samples from the Sacramento-San Joaquin Delta to resolve the inhibition of the PCR. The PCR cycling condition was performed as follows: initial denaturation step of 95°C for 5 min, 40 cycles of 95°C for 30 s, 50°C for 30 s, and 72°C for 2 min 30 s, followed by a final extension step at 72°C for 10 min and then held at 4°C. The PCR product was separated on 1% agarose gel and observed by a transilluminator after staining with 0.5 μg/ml ethidium bromide for 20 min.

**Table 4:** **Primers used for amplifying 16S ribosomal RNA gene sequences from algal samples from Sacramento-San Joaquin Delta and Clear Lake**

Target (size)	Primer	Sequence (5′ - 3′)	Reference
16S rDNA-ITS (1.5- 2 kb)	pA	AGAGTTTGATCCTGGCTCAG	Edwards et al. 1989
	B23S	CTTCGCCTCTGTGTGCCTAGGT	Lepere et al. 2000
16S rDNA (1.5 kb)	CYA108F	ACGGGTGAGTAACRCGTRA	Urbach et al. 2001
	CYA16S SCYR	CTTCAYGYAGGCGAGTTGCAGC	
16S rDNA for sequencing	AlgaeIDSqF4	CGTGCCAGCAGCCGCGGTAATACG	This study

The DNA bands at the expected size (1.5-2 kb) were excised from the gel and extracted using QIAquick II extraction kit (Qiagen). The eluted DNA was ligated into pGEM-T Easy vector (Promega BioSciences) that was used to transform *Escherichia coli* DH5α competent cells (Invitrogen). The length of the inserted DNA fragment was verified by running a PCR on colonies carrying the plasmid. The PCR cocktail (50 μl) contained 200 μM each of dNTP, 1.5 mM of MgCl_2_, 40 pmol of M13 forward and reverse primers, 0.5 unit Platinum Taq DNA polymerase (Invitrogen) and 10X buffer at 1/10 the volume of the reaction. The PCR cycling condition was the same as above except for the annealing temperature at 55°C. Clones carrying the inserted fragment size of 1.5 to 2 kb with variable length were chosen for plasmid extraction and sequencing. Twenty clones were analyzed for each of the algal samples except for the sample CL3(6) from which additional 30 clones were submitted for sequencing because various types of cyanobacterial sequences were observed from the first 20 clones. The plasmid was extracted using QIAprep Spin Mini Kit (Qiagen) according to the manufacturer’s instruction. The sequence of the inserted DNA fragment was determined from both ends using M13 forward and reverse primers in addition to the primer that we designed (AlgaeIDSqF4) for sequencing the middle fragments (Table 4). The samples were submitted to Davis Sequencing (http://www.davissequencing.com/) for sequencing reactions using an ABI 377 automated DNA sequencer (Applied Biosciences). The obtained sequences from each clone were processed to correct ambiguous bases and to remove vector and primer sequences; a consensus sequence was generated using Geneious software ver. 5.0.3 (Drummond et al. 2011).

The entire sequence of the 16S rDNA-ITS region was used for defining Operational Taxonomic Units (OTUs) using UCLUST ver. 1.2.22 with a threshold of 98.5% (Edgar 2010). A representative sequence for each of the OTU cluster was selected as a genotype and was used for similarity search using BLASTN (Altschul et al. 1990). The sequences showing similarity to cyanobacteria with potential ability to produce toxins were selected for further analysis.

The phylogenetic trees were constructed to distinguish species that share nearly identical 16S rDNA sequences with a few base pair differences such as those observed between *Aphanizomenon* and *Dolichospermum*, and between *Limnoraphis* and closely related species such as *Arthrospira* and *Lyngbya*. The 16S rDNA sequences, approximately 1.4 kb covering almost the entire sequence but not including the ITS regions, were used for phylogenetic analysis. The sequences used for phylogenetic tree analysis were taken from other studies as listed in Additional File 1 (Lehtimäki et al. 2000; Lyra et al. 2001; Gugger et al. 2002; Rajaniemi et al. 2005; Engene et al. 2011). Multiple alignments were generated by MUSCLE ver. 3.8.31 (Edgar 2004). The phylogenetic trees were generated by MrBayes program ver. 3.2 for 16S rDNA sequences using Markov chain Monte Carlo method with the following settings: Ngen = 10000000, Nchain = 4, Temp = 0.5, Stopval = 0.01, Samplefreq = 50, Printfreq = 1000 (Ronquist et al. 2012). The General Time Reversible model with a proportion of invariable sites and a gamma-shaped distribution of rates was selected by jModeltest ver. 2.1.4 (Darriba et al. 2012) as the best model for the datasets for the family Nostocaceae (*Aphanizomenon, Cuspidothrix*, and *Dolichospermum*) and Oscillatoriaceae (*Anthrospira*, *Limnoraphis*, and *Lyngbya*). *Nodularia* sp. (strain PCC7804) or *Plectonema wollei* (strain JW-2010c) was used as the outgroup for the phylogenetic tree of Nostocaceae (Figure 1) or Oscillatoriaceae (Figure 2), respectively. FigTree ver. 1.4 was used for depicting the phylogenetic trees (http://tree.bio.ed.ac.uk/software/figtree/).

## Authors’ information

TK, DVB, SJT are members of the Aquatic Health Program at UC Davis, School of Veterinary Medicine: http://www.vetmed.ucdavis.edu/aquatic_health/index.cfm.

## Abbreviations

CyanoHABs: Cyanobacterial blooms
ITS: Internal transcribed spacer
OTU: Operational taxonomic unit
rDNA: Ribosomal RNA gene.
